# AGPAT9 suppresses cell growth, invasion and metastasis by counteracting acidic tumor microenvironment through KLF4/LASS2/V-ATPase signaling pathway in breast cancer

**DOI:** 10.18632/oncotarget.4074

**Published:** 2015-06-17

**Authors:** Shao-hua Fan, Yan-yan Wang, Zhi-yong Wu, Zi-feng Zhang, Jun Lu, Meng-qiu Li, Qun Shan, Dong-mei Wu, Chun-hui Sun, Bin Hu, Yuan-lin Zheng

**Affiliations:** ^1^ Key Laboratory for Biotechnology on Medicinal Plants of Jiangsu Province, School of Life Science, Jiangsu Normal University, Xuzhou, Jiangsu, China; ^2^ Department of Function Examination, The First People's Hospital of Xuzhou, Jiangsu, China; ^3^ Obstetrics and Gynecology Hospital, Fudan University, Shanghai, China

**Keywords:** breast cancer, AGPAT9, acidic tumor microenvironment, proliferation, invasion

## Abstract

Human 1-acylglycerol-3-phosphate O-acyltransferase 9 (*AGPAT9*) is the gene identified from adipose tissue in 2007. We found *AGPAT9* expression was significantly higher in poorly invasive MCF7 human breast cancer cells than the highly invasive MDA-MB-231 cells. AGPAT9 significantly inhibited the proliferation of breast cancer cells *in vitro* and *in vivo*. Live-cell imaging and transwell assays showed that AGPAT9 could significantly inhibit the migration and invasive capacities of breast cancer cells. The inhibitory effect of AGPAT9 on metastasis was also observed *in vivo* in lung metastasis model. AGPAT9 inhibited breast cancer cell proliferation, migration and invasion through, at least in part, suppressing the V-ATPase activity. In addition, increased *AGPAT9* expression in MCF-7/ADR cells could increase the chemosensitivity to doxorubicin (Dox). Our findings suggest that increasing *AGPAT9* expression may be a new approach that can be used for breast cancer treatment.

## INTRODUCTION

Breast cancer is the most frequent malignancy in women and the second-leading cause of cancer-related deaths [1]. Breast cancer progression depends not only on primary tumor growth but also on the ability of tumor cells to metastasize to distant sites.

The vacuolar-H^+^-ATPase (V-ATPase) is an important pH regulatory complex in tumor cells and positively correlated to cancer invasion and metastasis, it is required to mediate signaling pathways, such as the Wnt/β-catenin pathway [2, 3]. V-ATPase uses the energy produced by ATP hydrolysis to pump protons into the extracellular environment. The low pH of tumor extracellular microenvironment may induce the increased activation of degradative enzymes, such as matrix metalloproteinases (MMPs). Moreover, low extracellular pH may promote the degradation and remolding of extracellular matrix (ECM) through proteolytic enzyme activation, thus contributing to cancer invasion and metastasis [4, 5]. V-ATPases are overexpressed in many types of metastatic cancers and positively correlated to their invasion and metastasis [5]. In breast cancer cells, the abundance of V-ATPase on the plasma membrane correlates with an invasive phenotype [6]. Furthermore, V-ATPase inhibitors reduce cell migration in cancer cells with high levels of plasma membrane V-ATPase [7, 8]. There is evidence that the inhibition of V-ATPase function via knockdown of *ATP6V0C* (ATPase, H^+^ transporting, lysosomal 16kDa, V0 subunit c) expression could effectively suppress cancer metastasis by the decrease of proton extrusion and the down-regulation of protease activity [9].

*LASS2* (*Homo sapiens* longevity assurance homolog 2 of yeast LAG1), which is also called *CERS2* (Ceramide synthase 2), is the gene identified from a human liver cDNA library and binds to ATP6V0C [10]. Our previous studies have shown that LASS2 was involved in chemotherapeutic outcomes and low *LASS2* expression may predict chemoresistance [11]. In addition, we also found higher expression of *LASS2* in the breast cancer patients was associated with fewer lymph node metastases [12].

*KLF4* (Kruppel-like factor 4), which is also called *EZF* (Epithelial zinc finger protein), is a transcription factor that participates in both tumor suppression and oncogenesis [13]. Transient adenoviral expression of *KLF4* in the 4T1 orthotopic mammary cancer model significantly attenuated primary tumor growth as well as micrometastases to the lungs and liver [14]. Overexpression of *KLF4* in the highly metastatic MDA-MB-231 breast tumor cell line was sufficient to restore E-cadherin expression and suppress migration and invasion [15]. Knockdown of *KLF4* in MCF7 cells elevated the growth rate of these cells in the presence of estrogen [13].

*AGPAT9* (1-acylglycerol-3-phosphate O-acy ltransferase 9), which is also called *LPCAT1* (lysophosphatidylcholine acyltransferase 1) [16–18], is a key enzyme for catalyzing the conversion of glycerol-3-phosphate to lysophosphatidic acid in the synthesis of triacylglycerol [19]. Until recently, AGPAT9 was cloned from adipose tissue. AGPAT9 is highly expressed in the lung and spleen, followed by leukocyte, omental adipose tissue, and placenta [20]. AGPAT9 has physiological roles in non-inflammatory platelet-activation factor remodeling pathway [21] and in retinal photoreceptor homeostasis [22]. Only recently, some researchers suggested that AGPAT9 maybe correlate with cancer risk [23, 24]. In this study, we found that *AGPAT9* expression was markedly different between MCF7 (poorly invasive breast cancer cells) and MDA-MB-231 (highly invasive breast cancer cells). We further elucidated the molecular mechanism of *AGPAT9* involved in breast cancer progression by the *in-vitro* assays and the *in-vivo* experiments.

## RESULTS

### Expression analysis of AGPAT9 in breast cancer cells

**Figure 1:**
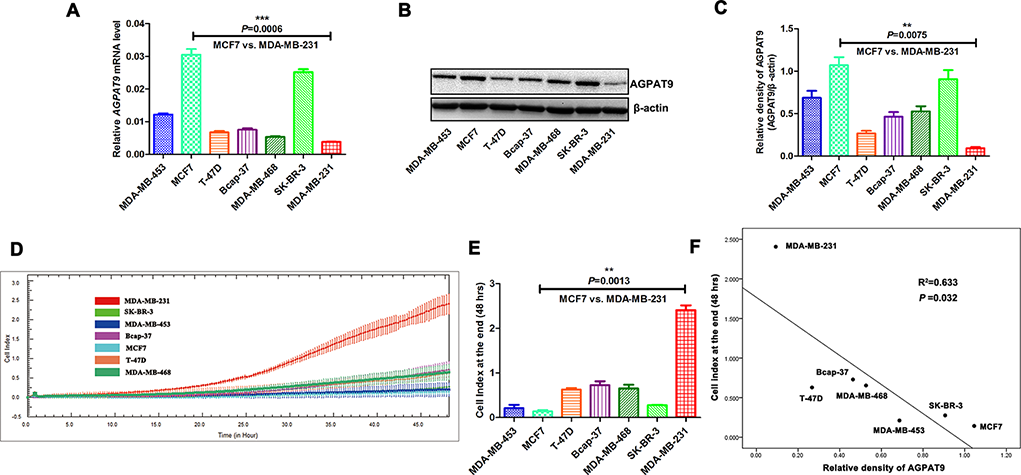
Association between *AGPAT9* expression and tumor invasion The expression levels of *AGPAT9* in various breast cancer cell lines were determined by quantitative real-time PCR **A.** and Western Blotting **B. C.** Relative density analysis of the AGPAT9 protein bands. The relative density is expressed as the ratio AGPAT9/β-actin. **D.** Real time invasion analysis of seven breast cancer cell lines. The methods were described in MATERIALS & METHODS. Invasion was monitored for 48 h in the xCELLigence DP system. The cell index was measured every 15 minutes. The rate of change of cell index as a function of time was calculated as a measure of invasive activity. **E.** The cell index at the end (48 hrs) is shown as a bar chart. **F.** Association between *AGPAT9* expression and tumor invasion in seven breast cancer cell lines.

To elucidate the role of AGPAT9 in breast cancer, we first examined the mRNA (Figure 1A) and protein (Figure 1B) expression of AGPAT9 in breast cancer cell lines. AGPAT9 was heterogeneously expressed in various breast cancer cells. MCF7 cells expressed relatively higher levels of AGPAT9 protein than other cells, and MDA-MB-231 cells expressed relatively lower levels of AGPAT9 protein (Figure 1C). To determine if there is a correlation between AGPAT9 protein levels and invasive abilities in breast cancer cell lines, we then examined the invasive ability of these cell lines using the RTCA xCELLigence system. Results showed that MCF7 cells are poorly invasive, and MDA-MB-231 cells are highly invasive (Figure 1D and 1E). These results are consistent with other reports [25, 26]. Intriguingly, across all cell lines tested, we found a significant inverse correlation between AGPAT9 protein levels and invasive abilities (*P* = 0.032; Figure 1F). We chose the relatively AGPAT9-highly-expressed cell line MCF7 (poorly invasive breast cancer cells) and the relatively AGPAT9-lowly-expressed cell line MDA-MB-231 (highly invasive breast cancer cells) for functional investigation.

### Effect of AGPAT9 on *in-vitro* proliferation

**Figure 2:**
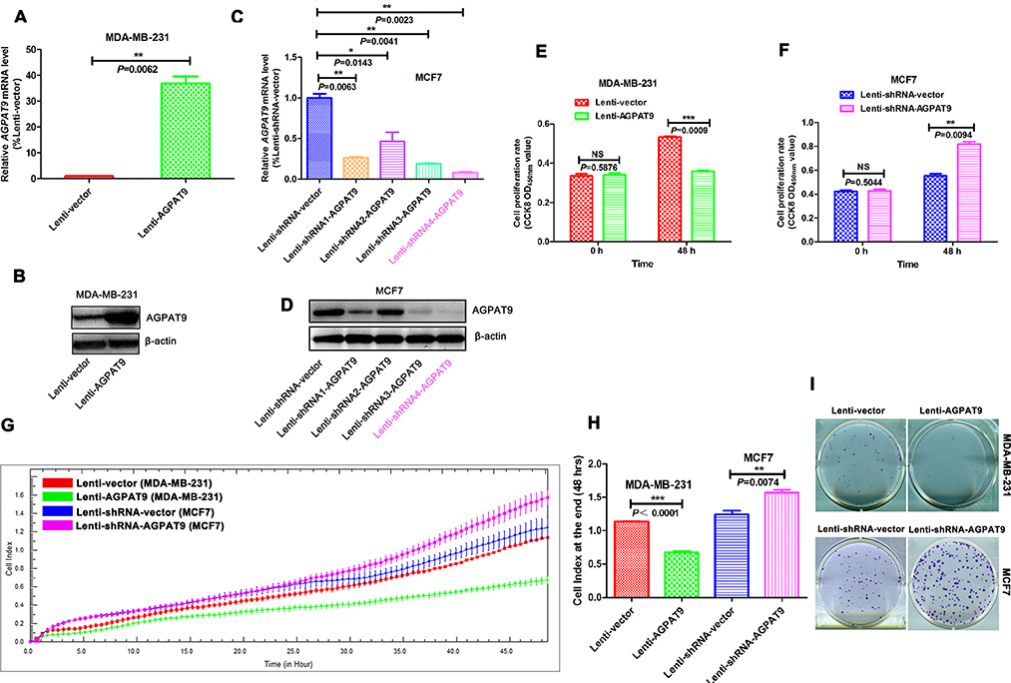
AGPAT9 inhibited breast cancer cell proliferation and clonogenicity *in vitro* Real time quantitative PCR **A.** and western blot **B.** analyses of *AGPAT9* expression in the AGPAT9 over-expression cell lines derived from MDA-MB-231 cells. Real time quantitative PCR **C.** and western blot **D.** analyses of *AGPAT9* expression in the AGPAT9 knock-down cell lines derived from MCF7 cells. The values shown are expressed as the mean ± S.E.M. The fragment marked with pink was used in the following functional experiments. In the CCK-8 cell proliferation assay, overexpression of *AGPAT9* in MDA-MB-231 cells significantly inhibited cell proliferation **E.** and *AGPAT9* knock-down in MCF-7 cells significantly increased cell proliferation **F.** Proliferation was monitored for 48 h in the xCELLigence DP system **G.** The cell index was measured every 30 minutes. The rate of change of cell index as a function of time was calculated as a measure of proliferation activity. **H.** The cell index at the end (48 hrs) is shown as a bar chart. **I.** *AGPAT9* over-expression inhibited and *AGPAT9*-shRNA enhanced cell foci formation ability. *P < 0.05; **P < 0.01; ***P < 0.001.

We established stable cell lines transduced by a lentivirus carrying the *AGPAT9* gene or no insert (vector control), which were designated as Lenti-AGPAT9 and Lenti-vector, respectively, in the breast cancer cell line MDA-MB-231 (Figure 2A and 2B). We also established stable cell lines transduced by a lentivirus carrying *AGPAT9*-short hairpin RNA (shRNA) or vector control, which was designated as Lenti-shRNA-AGPAT9 and Lenti-shRNA-vector, respectively, in the breast cancer cell line MCF-7 (Figure 2C and 2D). In the CCK-8 cell proliferation assay, overexpression of *AGPAT9* in MDA-MB-231 cells significantly inhibited cell proliferation with 48 h (*P* = 0.0009; Figure 2E), and *AGPAT9* knock-down in MCF-7 cells significantly increased cell proliferation with 48 h (*P* = 0.0094; Figure 2F). Furthermore, we used the xCELLigence system to analyze cell proliferation in real time (Figure 2G). Results showed overexpression of *AGPAT9* in MDA-MB-231 cells significantly inhibited cell proliferation with 48 h (*P* < 0.0001; Figure 2H), and *AGPAT9* knock-down in MCF-7 cells significantly increased cell proliferation with 48 h (*P* = 0.0074; Figure 2H). These are similar to the results of CCK-8 assay. Moreover, colony formation assay showed that enforced expression of *AGPAT9* resulted in a significant decrease in colony numbers in MDA-MB-231 cells compared with the vector controls, and reduced expression of *AGPAT9* resulted in a significant increase in colony numbers in MCF7 cells compared to control (Figure 2I).

### Effect of AGPAT9 on *in-vitro* migration and invasion

**Figure 3:**
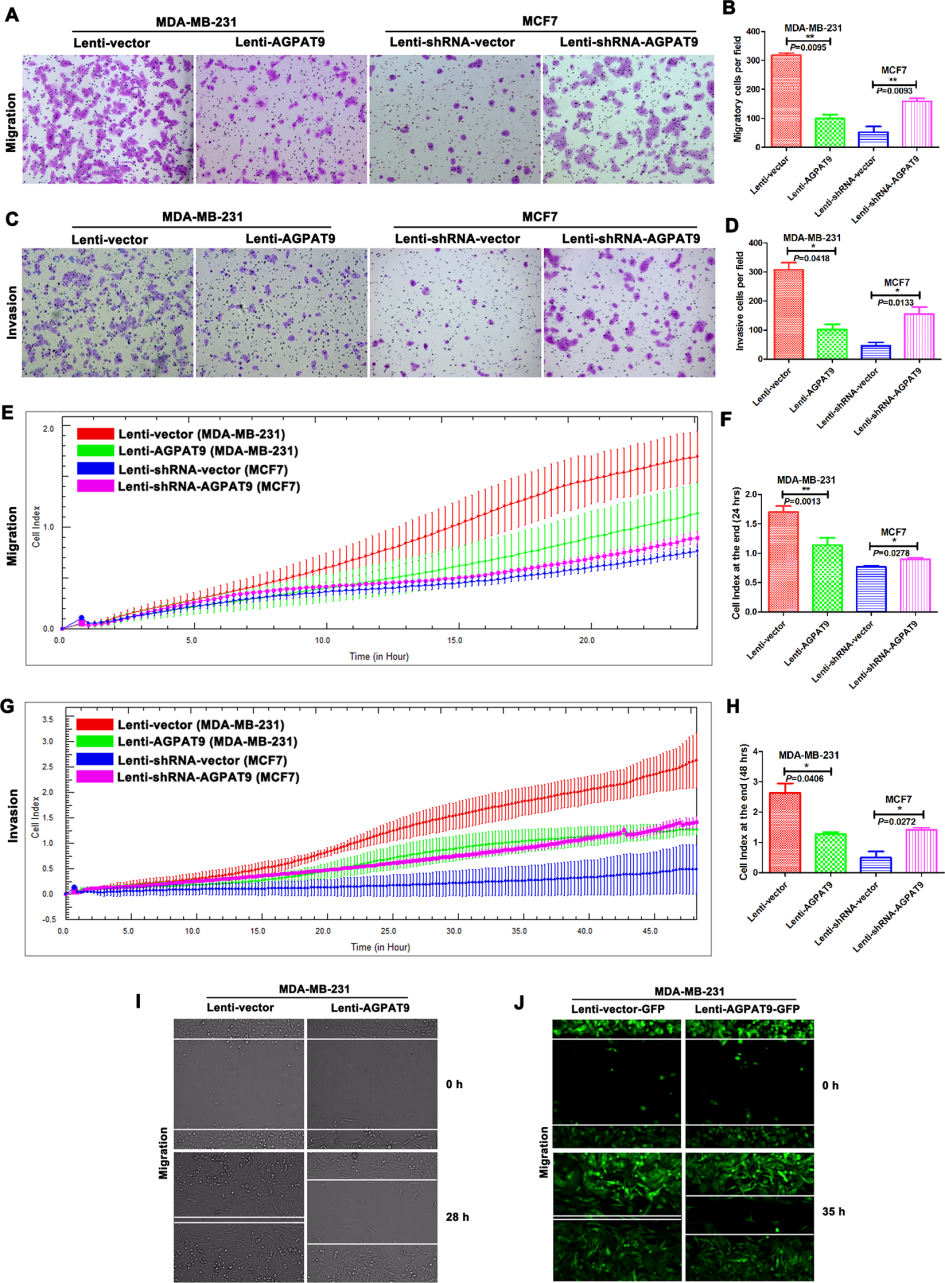
AGPAT9 inhibited breast cancer cell migration and invasion Transwell assays showed that overexpression of *AGPAT9* inhibited the migration **A, B.** and invasion **C, D.** rate of MDA-MB-231 cells, and knockdown of *AGPAT9* enhanced the migration (A, B) and invasion (C, D) rate of MCF7 cells. Migration **E.** or invasion **G.** was monitored for 24 h or 48 h in the xCELLigence DP system. The cell index was measured every 15 minutes. The rate of change of cell index as a function of time was calculated as a measure of migration or invasion activity. The cell index at the end (24 hrs or 48 hrs) is shown as a bar chart **F, H**. The migration of cells into the wound was monitored in multiple wells using a CellVoyager CV1000 confocal scanner system. The images were acquired every 0.5 hour for 28 hours (see Supplementary Movies S1 and S2) or 35 hours (see Supplementary Movies S3 and S4). The images shown represent 0 hour and 28 hours **I.** The distance between the two edges of the scratch in the Lenti-AGPAT9 well was obviously greater than that of the control. The images shown represent 0 hour and 35 hours **J.** The distance between the two edges of the scratch in the Lenti-AGPAT9-GFP well was obviously greater than that of the control. *P < 0.05; **P < 0.01.

Transwell assays without Matrigel demonstrated that overexpression of *AGPAT9* could significantly inhibit migration of MDA-MB-231 cells when compared with vector groups (*P* = 0.0095; Figure 3A and 3B), and *AGPAT9* knockdown in MCF7 cells significantly increased the migration capacity (*P* = 0.0093; Figure 3A and 3B). Transwell assays with Matrigel (Catalog Number: 356234, Protein Concentration: 8.95 mg/ml, BD Biosciences, MA) showed that overexpression of *AGPAT9* could significantly inhibit the invasive capacity of MDA-MB-231 cells when compared with the control cells (*P* = 0.0418; Figure 3C and 3D), and *AGPAT9* knockdown in MCF7 cells significantly increased the invasion capacity (*P* = 0.0133; Figure 3C and 3D). Furthermore, we used the xCELLigence system to analyze cell migration and invasion in real time. Results showed overexpression of *AGPAT9* in MDA-MB-231 cells significantly inhibited cell migration with 24 h (*P* = 0.0013; Figure 3E and 3F), and *AGPAT9* knock-down in MCF-7 cells significantly increased cell migration with 24 h (*P* = 0.0278; Figure 3E and 3F). The results also showed overexpression of *AGPAT9* in MDA-MB-231 cells significantly inhibited cell invasion with 48 h (*P* = 0.0406; Figure 3G and 3H), and *AGPAT9* knock-down in MCF-7 cells significantly increased cell invasion with 48 h (*P* = 0.0272; Figure 3G and 3H). These are similar to the results of Transwell assay. To further confirm this observation, we also determined the migration ability of breast cancer cells in the condition of *AGPAT9* overexpression using a confocal scanner system. The results showed that AGPAT9 significantly decreased the migration of MDA-MB-231 cells compared with the vector groups (Figure 3I and 3J; Supplementary Movies S1–S4).

### AGPAT9 inhibits cell proliferation, migration and invasion by up-regulating *LASS2* expression

**Figure 4:**
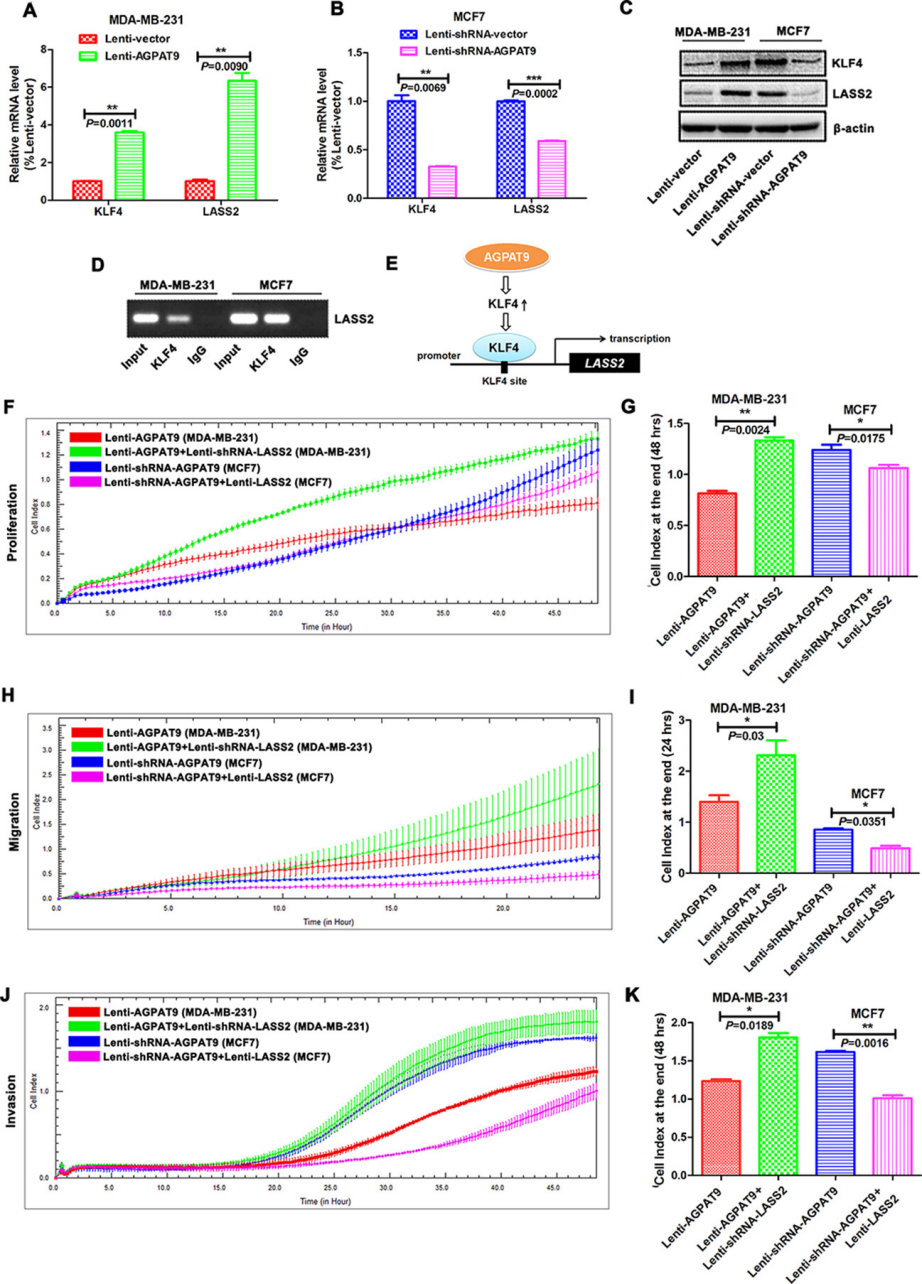
AGPAT9 overexpression resulted in increased expression of *LASS2* The real-time quantitative RT–PCR and western blot assays showed that enforced expression of *AGPAT9* resulted in a significant increase in KLF4 and LASS2 mRNA **A.** and protein **C.** level in MDA-MB-231 cells, and reduced expression of *AGPAT9* resulted in a significant decrease in KLF4 and LASS2 mRNA **B.** and protein (C) level in MCF7 cells. **D.** ChIP assay was performed with antibody against KLF4 or control IgG in MDA-MB-231 and MCF7 cells. The immunoprecipitated DNA was analyzed by PCR followed by agarose gel electrophoresis. **E.** Schematic diagram. RTCA assay showed that reduced expression of *LASS2* in Lenti-AGPAT9 (MDA-MB-231) cells could significantly increase cell proliferation **F, G.** migration **H, I.** and invasion **J, K.** compared with the Lenti-AGPAT9 (MDA-MB-231) cells, and enforced expression of *LASS2* in Lenti-shRNA-AGPAT9 (MCF7) cells could significantly inhibit cell proliferation (F, G), migration (H, I) and invasion (J, K) compared with the Lenti-shRNA-AGPAT9 (MCF7) cells. *P < 0.05; **P < 0.01; ***P < 0.001.

The real-time quantitative RT–PCR assay showed that enforced expression of *AGPAT9* resulted in a significant increase in *KLF4* (*P* = 0.0011; Figure 4A) and *LASS2* (*P* = 0.0090; Figure 4A) mRNA level in MDA-MB-231 cells compared with the vector controls, and reduced expression of *AGPAT9* resulted in a significant decrease in *KLF4* (*P* = 0.0069; Figure 4B) and *LASS2* (*P* = 0.0002; Figure 4B) mRNA level in MCF7 cells compared to control. Similar results were obtained by western blot analysis (Figure 4C).

Next, we explored whether LASS2 was a transcriptional target of KLF4. Chromatin immunoprecipitation (CHIP) assay showed that KLF4 directly bound to the promoter region of LASS2 in MDA-MB-231 or MCF7 cells (Figure 4D). The results demonstrate that the expression of *LASS2* is transcriptionally activated by KLF4 and *LASS2* is a target gene of KLF4 (Figure 4D and 4E). Intriguingly, RTCA proliferation assay showed that reduced expression of *LASS2* in Lenti-AGPAT9 (MDA-MB-231) cells could significantly increase cell proliferation compared with the Lenti-AGPAT9 (MDA-MB-231) cells (*P* = 0.0024; Figure 4F and 4G), and enforced expression of *LASS2* in Lenti-shRNA-AGPAT9 (MCF7) cells could significantly inhibit cell proliferation compared with the Lenti-shRNA-AGPAT9 (MCF7) cells (*P* = 0.0175; Figure 4F and 4G). Furthermore, RTCA migration assay showed that reduced expression of *LASS2* in Lenti-AGPAT9 cells could significantly increase cell migration compared with the Lenti-AGPAT9 cells (*P* = 0.03; Figure 4H and 4I), and enforced expression of *LASS2* in Lenti-shRNA-AGPAT9 cells could significantly inhibit cell migration compared with the Lenti-shRNA-AGPAT9 cells (*P* = 0.0351; Figure 4H and 4I). In addition, RTCA invasion assay showed that reduced expression of *LASS2* in Lenti-AGPAT9 cells could significantly increase cell invasion compared with the Lenti-AGPAT9 cells (*P* = 0.0189; Figure 4J and 4K), and enforced expression of *LASS2* in Lenti-shRNA-AGPAT9 cells could significantly inhibit cell invasion compared with the Lenti-shRNA-AGPAT9 cells (*P* = 0.0016; Figure 4J and 4K). Collectively, these data suggest that AGPAT9 inhibits breast cancer proliferation, migration and invasion through, at least in part, up-regulating the mRNA and protein levels of LASS2.

### Effect of AGPAT9 on the V-ATPase activity, pH_e_ and pH_i_

**Figure 5:**
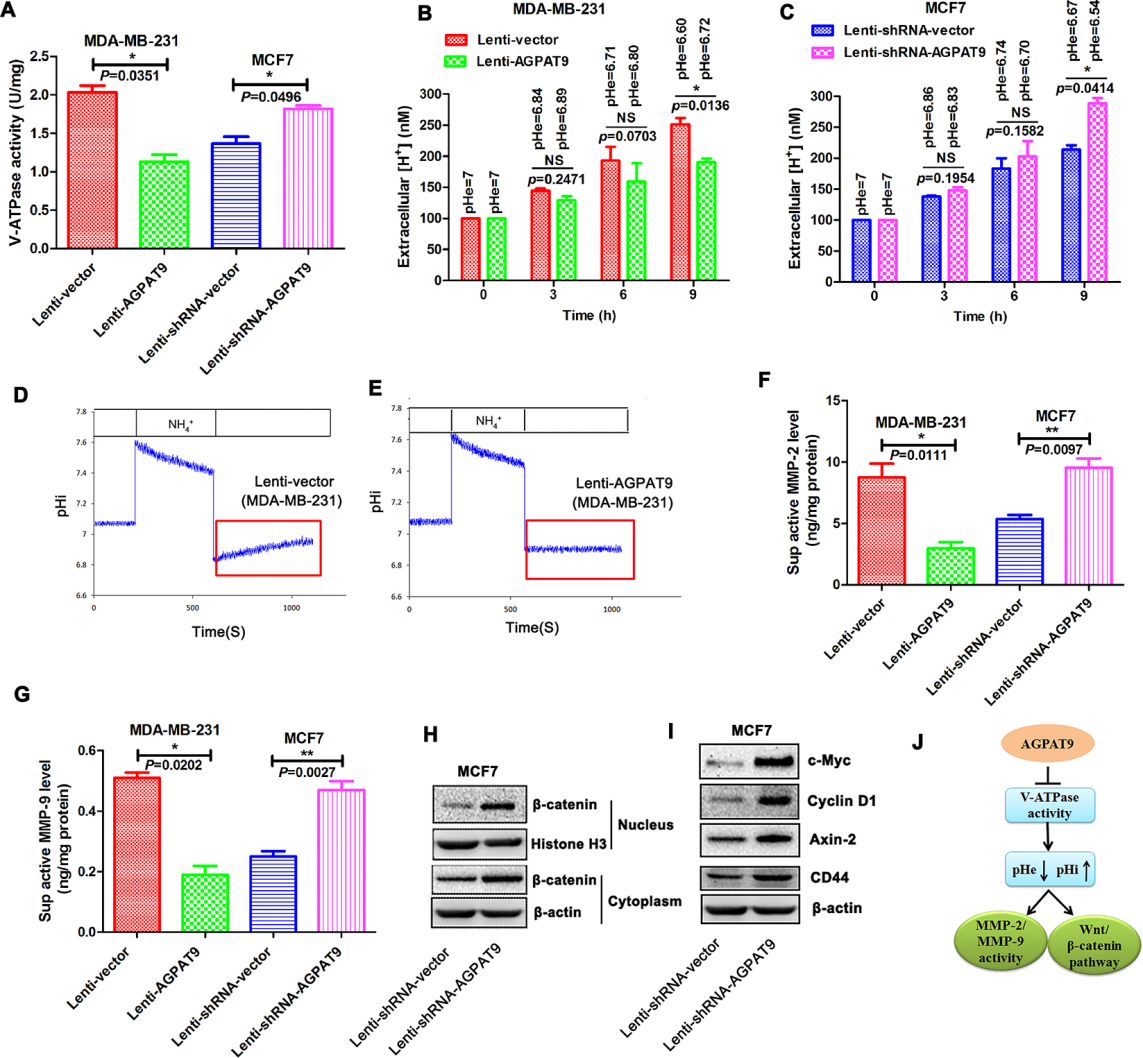
Effect of AGPAT9 on the V-ATPase activity, MMP-2 activity, MMP-9 activity and Wnt/β-catenin pathway **A.** Activity of V-ATPase in Lenti-AGPAT9 and Lenti-shRNA-AGPAT9 cells. **B.** Proton secretion was decreased in the Lenti-AGPAT9 cells compared with the Lenti-vector. **C.** Proton secretion was increased in the Lenti-shRNA-AGPAT9 cells compared with the Lenti-shRNA-vector. **D, E.** pH_i_ recovery analysis. The cells were pulsed with 40 mmol/NH_4_Cl and acid loaded by exposure to a Na^+^ and NH_4_^+^-free solution. The pH_i_ in Lenti-vector cells (D) recovered from the acid load. Lenti-AGPAT9 (E) did not recover to the baseline (indicated in red). The resting pH_i_ in Lenti-AGPAT9 cells decreased compared with Lenti-vector cells because of the defect of V-ATPase activity in Lenti-AGPAT9 cells. Active MMP-2 **F.** and active MMP-9 **G.** levels in culture supernatants were measured using the enzyme-linked immunosorbent assay (ELISA). Reduced expression of *AGPAT9* in MCF7 cells could result in an obvious increase in β-catenin protein level in both nucleus and cytoplasm compared to control **H.** The expression of Wnt/β-catenin pathway target genes, *c-Myc*, *Cyclin D1*, *Axin-2* and *CD44* were significantly increased in the Lenti-shRNA-AGPAT9 (MCF7) cells when compared with control cells **I. J.** Schematic diagram. *P < 0.05; **P < 0.01.

In this study, we found that AGPAT9 could significantly increase the mRNA and protein levels of LASS2. In previous study, we reported that LASS2 could inhibit the activity of V-ATPase proton pump through binding to ATP6V0C, the subunit of V-ATPase proton pump [11, 27]. Therefore, we examined the influence of AGPAT9 on the V-ATPase activity. Overexpression of *AGPAT9* in MDA-MB-231 cells significantly reduced the V-ATPase activity (*P* = 0.0351; Figure 5A), and *AGPAT9* knockdown in MCF7 cells significantly increased the V-ATPase activity (*P* = 0.0496; Figure 5A).

V-ATPases are involved in maintaining a relatively neutral intracellular pH and an acidic extracellular pH, through pumping protons into extracellular environment [11]. In this study, the process of proton extrusion was investigated by detecting the proton concentration in the medium with pH-sensitive BCECF. As shown in Figure 5B, the proton secretion of Lenti-AGPAT9 (MDA-MB-231) was notably reduced at 9 h compared with that of Lenti-vector (MDA-MB-231) (*P* = 0.0136; Figure 5B). Furthermore, the proton secretion of Lenti-shRNA-AGPAT9 (MCF7) was significantly increased compared with that of Lenti-shRNA-vector (MCF7) cells (*P* = 0.0414; Figure 5C). The pH_i_ (intracellular pH) recovery from intracellular acidification induced by NH_4_Cl prepulse is shown in Figure 5D–5E. When exposed to NH_4_Cl, pH_i_ increased rapidly and then decreased gradually. After the removal of NH_4_Cl, pH_i_ dropped rapidly. In the following procedure, the pH_i_ of Lenti-vector cells recovered (Figure 5D), whereas it hardly recovered in Lenti-AGPAT9 cells (Figure 5E). The results indicate that overexpression of *AGPAT9* results in inhibition of pH_i_ recovery due to AGPAT9 suppressing the function of V-ATPase in Lenti-AGPAT9 cells.

### Effect of AGPAT9 on the MMP-2 activity, MMP-9 activity and Wnt/β-catenin pathway

The supernatant of cultured cells was collected and the activities of MMP-2 and MMP-9 were assayed with MMP-2/MMP-9 Activity Assay kit. Intriguingly, overexpression of *AGPAT9* in the MDA-MB-231 cells could significantly decrease the active MMP-2 level in the supernatant of cultured cells (*P* = 0.0111; Figure 5F). Knockdown of *AGPAT9* in the MCF7 cells could significantly increase the active MMP-2 level in the supernatant of cultured cells (*P* = 0.0097; Figure 5F). Our results also showed overexpression of *AGPAT9* in the MDA-MB-231 cells could significantly decrease the active MMP-9 level in the supernatant of cultured cells (*P* = 0.0202; Figure 5G). Knockdown of *AGPAT9* in the MCF7 cells could significantly increase the active MMP-9 level in the supernatant of cultured cells (*P* = 0.0027; Figure 5G). These results showed that AGPAT9 have significant effects on the active MMP-2 and MMP-9 levels in the supernatant.

The Wnt/β-catenin pathway is one of the most important signal transduction pathways in breast cancer cell growth. The accumulation of β-catenin in the cells, especially in the nucleus, is considered as an important indicator of the activation of Wnt/β-catenin pathway [28]. Our results showed that reduced expression of *AGPAT9* in MCF7 cells could result in an obvious increase in β-catenin protein level in both nucleus and cytoplasm compared to control (Figure 5H). Furthermore, expression of Wnt/β-catenin pathway target genes, *c-Myc*, *Cyclin D1*, *Axin-2* and *CD44* were significantly increased in the Lenti-shRNA-AGPAT9 (MCF7) cells when compared with control cells (Figure 5I). Collectively, these data suggest that AGPAT9 influences breast cancer proliferation through, at least in part, regulating the Wnt/β-catenin pathway (Figure 5J).

### Effect of AGPAT9 on *in vivo* proliferation and metastasis

**Figure 6:**
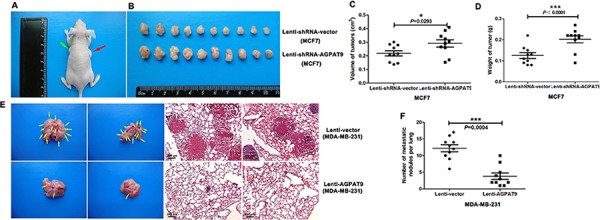
AGPAT9 inhibited tumorigenic and spontaneous lung metastatic capabilities of breast cancer cells **A.** 17β-Estradiol pellets were subcutaneously implanted into the left shoulder area of mice (green arrow) 3 days before tumor cell injection. The red arrow shows the tumor. **B.** The photo of tumors isolated from sacrificed nude mice of the indicated groups. **C, D.** the volume and weight of the tumors. **E.** Representative H&E stained sections of the lung tissues isolated from mice that injected with Lenti-Vector or Lenti-AGPAT9 cells through the lateral tail vein, Arrow head points to the tumor focus formed in the lung. **F.** The numbers of metastases in the lungs were counted. Scale bars: 200 μm (E) *P < 0.05; ***P < 0.001.

To determine the *in vivo* effects of AGPAT9, we performed *in vivo* proliferation and metastasis study. The average size and weight of xenografts in the Lenti-shRNA-AGPAT9 (MCF7) group were dramatically larger and heavier than those of the control group (*P* = 0.0293 and *P* < 0.0001, respectively). (Figure 6A–6D). Furthermore, we injected Lenti-vector (MDA-MB-231) or Lenti-AGPAT9 (MDA-MB-231) cells into the lateral tail veins of nude mice (*n* = 10) and evaluated the metastatic growth of cells in the lung. After 100 days, the Lenti-AGPAT9 mice displayed a statistically significantly lower number of lung metastases than the control group mice (*P* = 0.0004; Figure 6E and 6F), indicative of extravasation and tumor growth in the lung. When lungs underwent hematoxylin and eosin staining, lung metastases were observed in all ten mice intravenously injected Lenti-vector cells (Figure 6E, up), whereas much less lung metastases were observed in the mice intravenously injected Lenti-AGPAT9 cells (Figure 6E, down).

### Effect of AGPAT9 on chemosensitivity

We found that the *AGPAT9* was significantly decreased at mRNA level (–240.368-fold, *P* = 0.0006, Supplementary Figure S1A) in the drug-resistant breast cancer cell line MCF7/ADR than in the drug-sensitive breast cancer cell line MCF7. Next, we established stable cell lines transduced by a lentivirus carrying the *AGPAT9* gene or no insert (vector control) in the MCF7/ADR cells. Overexpression of *AGPAT9* in MCF7/ADR cells significantly reduced the IC_50_ value for Dox (*P* = 0.0146; Supplementary Figure S1B). To investigate the influence of AGPAT9 on the subcellular distribution of Dox in the cells, *AGPAT9*-overexpressing cells and control cells were treated with Dox (Supplementary Figure S1C). After uptake by MCF7 cells, Dox was localized mostly in the nuclei, which is in line with the sensitivity of these cells to the cytotoxic effect of Dox. In contrast, Dox was localized in discrete granules in the cytosol and barely detectable in the nuclei of control and Lenti-vector (MCF7/ADR) cells. However, in Lenti-AGPAT9 (MCF7/ADR) cells, Dox was localized in the nuclei, as assayed by confocal fluorescence microscopy. These results indicate that enforced expression of *AGPAT9* in MCF7/ADR cells results in a clear nuclear targeting of Dox. In addition, we also determined the migration ability of breast cancer cells in the condition of *AGPAT9* overexpression using a confocal scanner system. The results showed that AGPAT9 significantly decreased the migration of MCF7/ADR cells compared with the vector groups (Supplementary Figure S2; Supplementary Movies S5 and S6).

## DISCUSSION

Evidences are accumulating that acidic tumor microenvironment is involved in cancer progression [29, 30]. The V-ATPase is the primary regulator of the tumor microenvironment, by means of proton extrusion to the extracellular medium. Several V-ATPase inhibitors have been reported to inhibit cancer cell proliferation, invasion and metastasis [8, 31].

In this study, we found that the mRNA level of *AGPAT9* in MDA-MB-231 cells (highly invasive breast cancer cells) was significantly lower than in MCF7 cells (poorly invasive breast cancer cells) (–8.06-fold; Figure 1A). Furthermore, we confirmed this differential expression at the protein level (–11.24-fold; Figure 1B and 1C). Intriguingly, across all breast cancer cell lines tested, we found a significant inverse correlation between AGPAT9 protein levels and invasive abilities (*P* = 0.032; Figure 1F). The *in-vitro* data showed overexpression of *AGPAT9* in MDA-MB-231 cells significantly inhibited cell proliferation, migration and invasion (Figures 2 and 3). The *in-vitro* data also showed *AGPAT9* knock-down in MCF7 cells significantly increased cell proliferation, migration and invasion (Figures 2 and 3). Similar results were obtained in *in-vivo* experiments (Figure 6). Furthermore, overexpression of *AGPAT9* in Bcap-37 cells significantly inhibited cell proliferation, migration and invasion, and *AGPAT9* knockdown in Bcap-37 cells significantly increased cell proliferation, migration and invasion (Supplementary Figure S3).

KLF4 inhibits breast cancer cell proliferation, migration and invasion [13, 15]. We found that enforced expression of *AGPAT9* resulted in a significant increase in *KLF4* mRNA level, and reduced expression of *AGPAT9* resulted in a significant decrease in *KLF4* mRNA level. Similar results were obtained by western blot analysis. Further study is underway to explore the mechanism that AGPAT9 increases expression of *KLF4*. Our previous study indicated that LASS2 inhibited cancer cell proliferation, migration and invasion [11, 27]. Furthermore, we found that the expression of *LASS2* is transcriptionally activated by KLF4 and *LASS2* is a target gene of KLF4. In previous study, we reported that LASS2 could inhibit the V-ATPase activity through binding to ATP6V0C, the c subunit of V-ATPase proton pump [11, 27]. Furthermore, we found that inhibition of V-ATPase activity by knocking down *ATP6V0C* expression in MDA-MB-231 cells significantly reduced the migration (Supplementary Figure S4; Supplementary Movies S7 and S8). These results suggest that AGPAT9 may inhibit malignant progression of breast cancer by inhibiting the V-ATPase activity through increasing the *LASS2* expression. To explore this possibility, we examined the influence of AGPAT9 on the V-ATPase activity, pH_e_ and pH_i_ in the breast cancers. The results showed that overexpression of *AGPAT9* could reduce the V-ATPase activity (Figure 5A), increase the pH_e_ (Figure 5B) and decrease the pH_i_ (Figure 5D and 5E) in MDA-MB-231 cells. Meanwhile, *AGPAT9* knockdown in MCF7 cells significantly increased the V-ATPase activity (Figure 5A).

The promoting effect of V-ATPase on cancer invasion mainly relies on its maintain acidic pH of extracellular microenvironment, which is related to the activation of many proteases involved in the digestion of ECM [9]. The pH-sensitive proteases include cathepsin (cathepsin B, D, etc.) and MMPs (MMP-2, MMP-9, MMP-3, etc.) [9, 32]. Intriguingly, *AGPAT9* overexpression in invasive MDA-MB-231 cells is associated with decreased activities of MMP-2 and MMP-9, whereas suppression of AGPAT9 had the opposite effects on MMPs in non-invasive MCF7 cells (Figure 5F and 5G). AGPAT9 inhibits breast cancer invasion through, at least in part, up-regulating the activities of MMP-2 and MMP-9 (Figure 5F and 5G). Very recently, some researchers found that V-ATPase was required to mediate Wnt/β-catenin signaling [2, 33, 34]. Wnt/β-catenin signaling is important for cell proliferation. Activation of the Wnt/β-catenin signaling stimulates cell growth. V-ATPase generates a proton gradient that is essential for LRP6 phosphorylation and hence β-catenin activation [2]. AGPAT9 inhibits breast cancer proliferation through, at least in part, regulating the Wnt/β-catenin pathway (Figure 5H and 5I).

Doxorubicin (Adriamycin) belongs to the family of anthracyclines. It is membrane permeable in its neutral form and relatively membrane impermeable when protonated [35]. Therefore, the acid pH_e_ of cancer cells can make Dox become protonated and hinder it from entering the cells [36]. Chemotherapeutic agents are distributed throughout the cytoplasm and nucleus of drug-sensitive cells. In contrast, in MDR cells, they accumulate only within discrete cytoplasmic organelles, and almost none is detectable in the nucleus, the target of anthracycline drugs [37, 38]. We found that the overexpression of *AGPAT9* increased the susceptibility to Dox cytotoxicity in MCF7/ADR cells. This effect might be caused by a significant increase in pH_e_, and more Dox entered the cells and stayed in the nuclei of cell (Supplementary Figure S1C).

**Figure 7:**
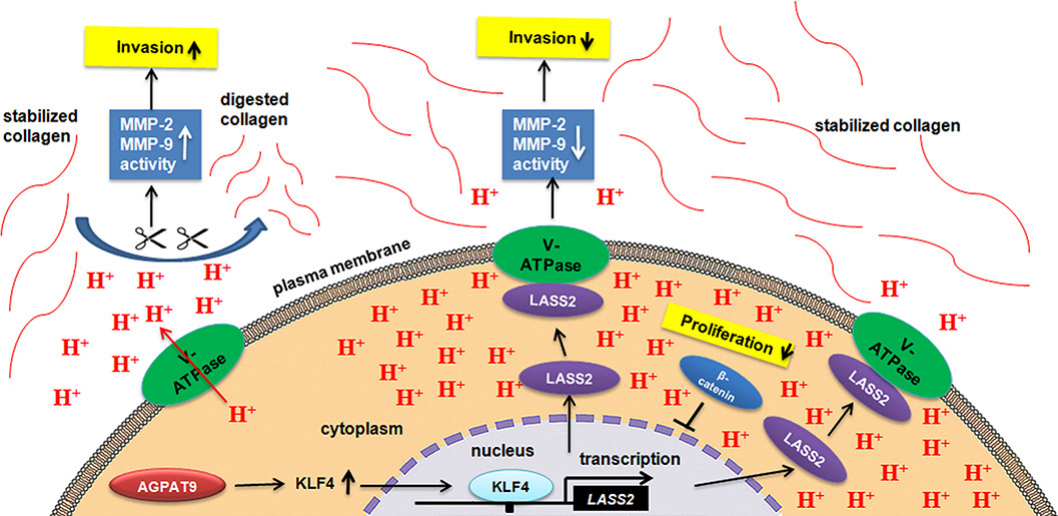
Effect of AGPAT9 on the Wnt/β-catenin pathway A hypothetical cascade pathway of the suppression of tumor cell invasion and proliferation by AGPAT9. AGPAT9 could significantly inhibit the V-ATPase activity and extracellular hydrogen ion concentration, and in turn the activation of secreted MMP-2/MMP-9, which ultimately suppressed tumor's invasion. AGPAT9 inhibited breast cancer proliferation through, at least in part, suppressing the V-ATPase activity and Wnt/β-catenin pathway.

In summary, we first delineate the molecular mechanism that AGPAT9 inhibits human breast cancer cells proliferation, invasion and metastasis (Figure 7). Our findings suggest that increasing *AGPAT9* expression may be a new approach that can be used for breast cancer treatment and imply that acidic tumor microenvironment may be considered as an important influential factor of antitumor therapeutic efficacy.

## MATERIALS AND METHODS

### Cell lines

Human breast cancer cell lines, T-47D, SK-BR-3, MDA-MB-468, MDA-MB-453, MDA-MB-231 and MCF7, were purchased from the American Type Culture Collection (ATCC, Manassas, VA). Human breast cancer cell line Bcap-37 was purchased from the Committee on Type Culture Collection of Chinese Academy of Sciences (Shanghai, China).

### Vector constructs and lentivirus production

The *AGPAT9* lentivirus expression vector was constructed by amplifying the coding sequence of human *AGPAT9* and cloning it into the LV5/GFP/Puro (Genepharma, Shanghai, China) or LV6/Puro vector (Genepharma, Shanghai, China). Oligonucleotides were synthesized to generate an annealing shRNA targeting the sequence of *AGPAT9* from position 606–626 (5′-GGGAACTCTGATCCAGTATA T-3′), 709–729 (5′-GGAAAGTGGCCACAGATAATG-3′), 758–778 (5′-GGACCT GCCTAATTACCTTCA-3′) or from 1204–1224 (5′-GGAGGAGAGAAGATAGGT ATT-3′). The fragments were cloned separately into pGLV3/H1/GFP/Puro vector (Genepharma, Shanghai, China) using the restriction sites *BamHI* and *EcoRI*.

The *LASS2* lentivirus expression and shRNA-expressing vector were detailed in our previous study [11]. The other plasmids or recombinant vectors used are shown in Supplementary Tables 1 and 2.

### Quantitative real-time PCR

Real-time PCR analyses were performed with SYBR *Premix Ex Taq* (TaKaRa). The sequences of primers used were as follows: *AGPAT9*: 5′-CACCGTGACCGACCTATTC-3′ and 5′-GCCCAGCGTCTGAGTTTT-3′; *LASS2*: 5′-GCCCAAGCAGGTGGAAGTAGAG-3′ and 5′-CCAGGGTTTA-TCCACAATGACG-3′.

### Chromatin immunoprecipitation assay

The assay was performed using the EZ-ChIP™ kit (#17–371, Millipore, Billerica, MA) according to the manufacturer's instructions. The following antibodies were utilized to immunoprecipitate crosslinked protein-DNA complexes: rabbit anti-KLF4 (sc-20691, Santa Cruz) and normal rabbit IgG (12–370, Millipore). The immunoprecipitated DNA was purified for PCR analyses with primers within the promoter of *LASS2*.

### Activity of V-ATPase

Assays were performed as described previously by us [11, 27].

### Measurement of extracellular pH and intracellular pH

Assays were performed as described previously by us [11, 27].

### Protein extraction and western blotting

Total protein was extracted from the homogenate of cells using the T-PER Tissue Protein Extraction Reagent (Thermo Scientific, #78510). The proteins were separated by SDS-PAGE and transferred to nitrocellulose membrane (Bio-Rad, Hercules, CA). The membrane was blocked with 5% non-fat milk and incubated with rabbit anti-AGPAT9 polyclonal antibody (pAb) (Sigma Aldrich) (1:1000), rabbit anti-KLF4 pAb (Santa Cruz Biotechnology) (1:1000), rabbit anti-LASS2 pAb (Santa Cruz Biotechnology) (1:1000), rabbit anti-β-catenin monoclonal antibody (mAb) (Cell Signaling Technology) (1:1000), rabbit anti-Histone H3 pAb (Santa Cruz Biotechnology) (1:1000), rabbit anti-c-Myc mAb (Cell Signaling Technology) (1:1000), rabbit anti-Cyclin D1 mAb (Cell Signaling Technology) (1:1000), rabbit anti-Axin-2 mAb (Cell Signaling Technology) (1:1000) or mouse anti-CD44 mAb (Cell Signaling Technology) (1:1000). The proteins were detected with enhanced chemiluminescence reagents (Pierce).

### Xenograft model and treatments

Two different mouse models were used to observe *in vivo* effect of AGPAT9 on breast cancer cells. For the subcutaneous model, 17β-estradiol pellets (NE-121, Innovative Research of America, Sarasota, FL) were subcutaneously implanted into the left shoulder area of mice 3 days before tumor cell injection. MCF7 cells (1 × 10^7^) with stable knockdown of *AGPAT9* or the control vector were suspended in 200 μl serum-free DMEM and subcutaneously injected into the right upper ﬂank of each mouse (ten per group, female BALB/c-nu/nu, 8 weeks old) [39, 40]. At day 11 after cell injection, mice were allocated to the Lenti-shRNA-vector group and Lenti-shRNA-AGPAT9 group. The tumors were measured using digital calipers every 3 to 4 days after they reached a volume of 100 mm^3^, and tumor volumes were calculated as described: V (cm^3^) = Width^2^ (cm^2^) × Length (cm) / 2. At the termination of the experiment, the mice were sacrificed by cervical dislocation, and the tumors were weighed immediately after dissection.

For lung metastasis experiments, MDA-MB-231 (1.5 × 10^6^) stably expressing *AGPAT9* or the control vector were suspended in 200 μl PBS and injected into the tail veins of each mouse (female BALB/c-nu/nu, 8 weeks old) [41]. Then, the animals were equally divided into two groups (ten per group): Lenti-vector group and Lenti-AGPAT9 group. At day 100 after cell injection, the mice were sacrificed by cervical dislocation, and their lungs were removed and subjected to hematoxylin and eosin (H&E) staining. Mice were manipulated and housed according to the protocols approved by the Committee on the Ethics of Animal Experiments of Jiangsu Normal University.

### Statistical analysis

The results are presented as the means and s.e.m. The data were subjected to Student's *t*-test (two-tailed; *P* < 0.05 was considered significant) unless otherwise specified (χ^2^ test, Pearson's correlation, and linear regression).

## SUPPLEMENTARY TABLES, FIGURES AND MOVIES


