# Supplementary material for: AGPAT9 suppresses cell growth, invasion and metastasis by counteracting acidic tumor microenvironment through KLF4/LASS2/V-ATPase signaling pathway in breast cancer
Source: Oncotarget. 2015 Jun 17;6(21):18406–17. doi: 10.18632/oncotarget.4074 (PMC4621899; doi:10.18632/oncotarget.4074)
Supplement: Supplementary file 1 [file oncotarget-06-18406-s001.pdf]

SUPPLEMENTARY TABLES, FIGURES AND MOVIES

Supplementary Table S1: The plasmids used for the construction and transfection

Plasmids	Description	Origin
pGLV3/H1/GFP/Puro	Plasmid contruction	Genepharma, Shanghai, China
LV5/GFP/Puro	Plasmid contruction	Genepharma, Shanghai, China
LV6/Puro	Plasmid contruction	Genepharma, Shanghai, China
PG-P1-VSVG	Lenti-virus production	Genepharma, Shanghai, China
PG-P2-REV	Lenti-virus production	Genepharma, Shanghai, China
PG-P3-RRE	Lenti-virus production	Genepharma, Shanghai, China
pLVTHM	Plasmid contruction	Addgene, Cambridge, MA
pWPXL	Plasmid contruction	Addgene, Cambridge, MA
psPAX2	Lenti-virus production	Addgene, Cambridge, MA
pMD2.G	Lenti-virus production	Addgene, Cambridge, MA

Supplementary Table S2: The sequences of the oligonucleotides for plasmids construct

Name	Sequence
Lenti-shRNA-vector	L: 5′-GATCCGTTCTCCGAACGTGTCACGTTTCAAGAGAA
(for AGPAT9)	CGTGACACGTTCGGAGAACTTTTTTG-3′
	R: 5′-AATTCAAAAAAGTTCTCCGAACGTGTCACGTTCT
	CTTGAAACGTGACACGTTCGGAGAACG-3′
Lenti-shRNA1-AGPAT9	L: 5′-GATCCGGGAACTCTGATCCAGTATATTTCAAGAGA
	ATATACTGGATCAGAGTTCCCTTTTTTG-3′
	R: 5′-AATTCAAAAAAGGGAACTCTGATCCAGTATATTCT
	CTTGAAATATACTGGATCAGAGTTCCCG-3′
Lenti-shRNA2-AGPAT9	L: 5′-GATCCGGAAAGTGGCCACAGATAATGTTCAAGAG
	ACATTATCTGTGGCCACTTTCCTTTTTTG-3′
	R: 5′-AATTCAAAAAAGGAAAGTGGCCACAGATAATGTC
	TCTTGAACATTATCTGTGGCCACTTTCCG-3′
Lenti-shRNA3-AGPAT9	L: 5′-GATCCGGACCTGCCTAATTACCTTCATTCAAGAGA
	TGAAGGTAATTAGGCAGGTCCTTTTTTG-3′
	R: 5′-AATTCAAAAAAGGACCTGCCTAATTACCTTCATCT
	CTTGAATGAAGGTAATTAGGCAGGTCCG-3′

(continued)

Name	Sequence
Lenti-shRNA4-AGPAT9	L: 5′-GATCCGGAGGAGAGAAGATAGGTATTTCAAGAGA
	ATACCTATCTTCTCTCCTCCTTTTTTG-3′
	R: 5′-AATTCAAAAAAGGAGGAGAGAAGATAGGTATTCT
	CTTGAAATACCTATCTTCTCTCCTCCG-3′
Lenti-LASS2	L: 5′- TATACGCGTATGCTCCAGACCTTGTA-3′
	R: 5′-GCTACTAGTTCAGTCATTCTTACGATGGT-3′
Lenti-shRNA-vector	L: 5′- CGCGTCCCCTTCTCCGAACGTGTCACGTTTCAAG
(for LASS2 or ATP6V0C)	AGAACGTGACACGTTCGGAGAATTTTTGGAAAT-3′
	R: 5′-CGATTTCCAAAAATTCTCCGAACGTGTCACGTT
	CTCTTGAAACGTGACACGTTCGGAGAAGGGGA-3′
Lenti-shRNA1-LASS2	L: 5′-CGCGTCCCCGGCCCAGTCTCCTCAAGAATTCAAG
	AGATTCTTGAGGAGACTGGGCCTTTTTGGAAAT-3′
	R: 5′-CGATTTCCAAAAAGGCCCAGTCTCCTCAAGAA
	TCTCTTGAATTCTTGAGGAGACTGGGCCGGGGA-3′
Lenti-shRNA2-LASS2	L: 5′-CGCGTCCCCAGTATTGGTACTACATGATTTCAA
	GAGAATCATGTAGTACCAATACTTTTTGGAAAT-3′
	R: 5′-CGATTTCCAAAAAAGTATTGGTACTACATGATT
	CTCTTGAAATCATGTAGTACCAATACTGGGGA-3′
Lenti-shRNA1-ATP6V0C	L: 5′-CGCGTCCCCTCGGCCTCTACGGTCTCATTTCAAGA
	GAATGAGACCGTAGAGGCCGATTTTTGGAAAT-3′
	R: 5′-CGATTTCCAAAAATCGGCCTCTACGGTCTCATTC
	TCTTGAAATGAGACCGTAGAGGCCGAGGGGA-3′
Lenti-shRNA2-ATP6V0C	L: 5′- CGCGTCCCCGTCCATCATCCCAGTGGTCTTCAAG
	AGAGACCACTGGGATGATGGACTTTTTGGAAAT-3′
	R: 5′- CGATTTCCAAAAAGTCCATCATCCCAGTGGTC
	TCTCTTGAAGACCACTGGGATGATGGACGGGGA-3′

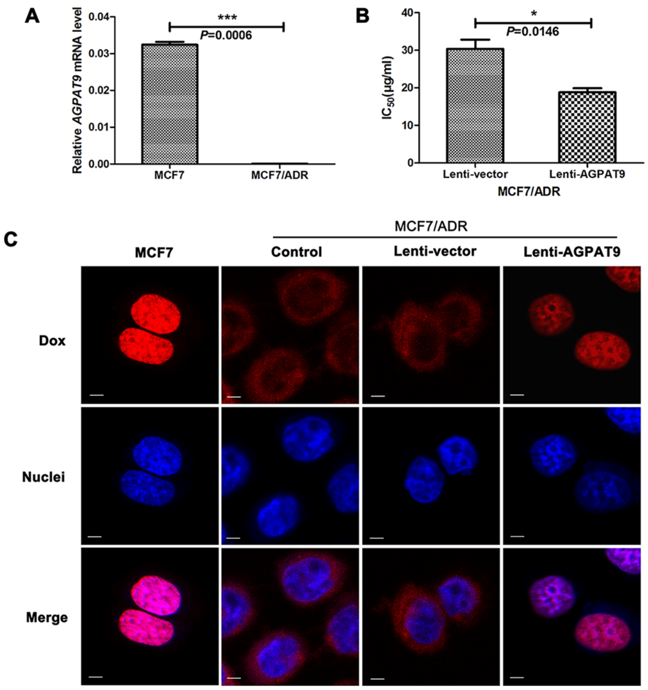

Supplementary Figure S1: Correlation analysis of *AGPAT9* expression and chemosensitivity. A. The relative mRNA levels of *AGPAT9* in drug-sensitive MCF7 and drug-resistant MCF7/ADR cells. **B.** The overexpression of *AGPAT9* in MCF7/ADR cells obviously decreased the IC_{50} value for Dox. **C.** The cells were visualized by confocal microscopy after sequentially loaded with Dox and Hoechst 33342. Red fluorescence indicates Dox; blue fluorescence indicates nuclei. Scale bars: 5 μm (C).

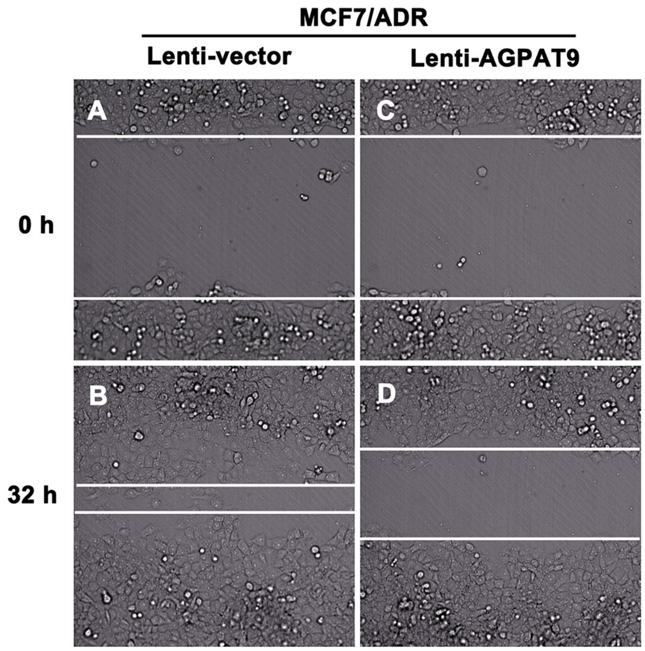

Supplementary Figure S2: Overexpression of *AGPAT9* inhibited the migration of MCF7/ADR cells. The migration of cells into the wound was monitored in multiple wells using a CellVoyager CV1000 confocal scanner system. The images were acquired every hour for 32 hours (see Supplementary Movies S5 and S6). The images shown represent 0 hour **A, C.** and 32 hours **B, D.** The distance between the two edges of the scratch in the Lenti-AGPAT9 well (D) was greater than that of the control (B).

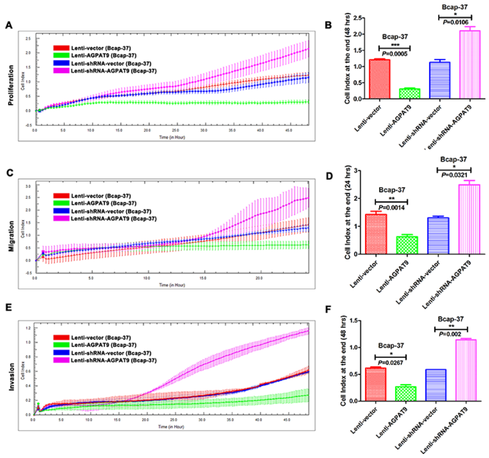

Supplementary Figure S3: Effect of overexpression or knockdown of *AGPAT9* on the proliferation, migration and invasion abilities of Bcap-37 cells. Proliferation was monitored for 48 h in the xCELLigence DP system **A.** The cell index was measured every 30 minutes. The rate of change of cell index as a function of time was calculated as a measure of proliferation activity. **B.** The cell index at the end (48 hrs) is shown as a bar chart. Migration **C.** or invasion **E.** was monitored for 24 h or 48 h in the xCELLigence DP system. The cell index was measured every 15 minutes. The rate of change of cell index as a function of time was calculated as a measure of migration or invasion activity. The cell index at the end (24 hrs or 48 hrs) is shown as a bar chart **D, F.**

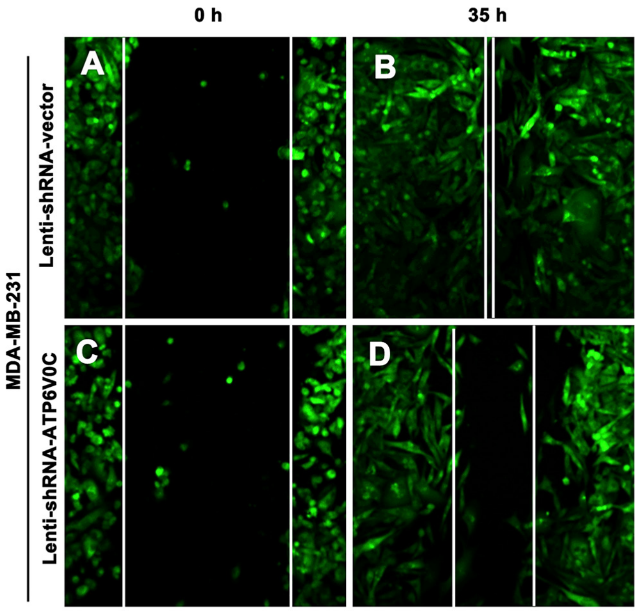

Supplementary Figure S4: Knockdown of *ATP6V0C* inhibited the migration of MDA-MB-231 cells. The migration of cells into the wound was monitored in multiple wells using a CellVoyager CV1000 confocal scanner system. The images were acquired every 0.5 hour for 35 hours (see Supplementary Movies S7 and S8). The images shown represent 0 hour **A, C.** and 35 hours **B, D.** The distance between the two edges of the scratch in the Lenti-shRNA-ATP6V0C well (D) was greater than that of the control (B).

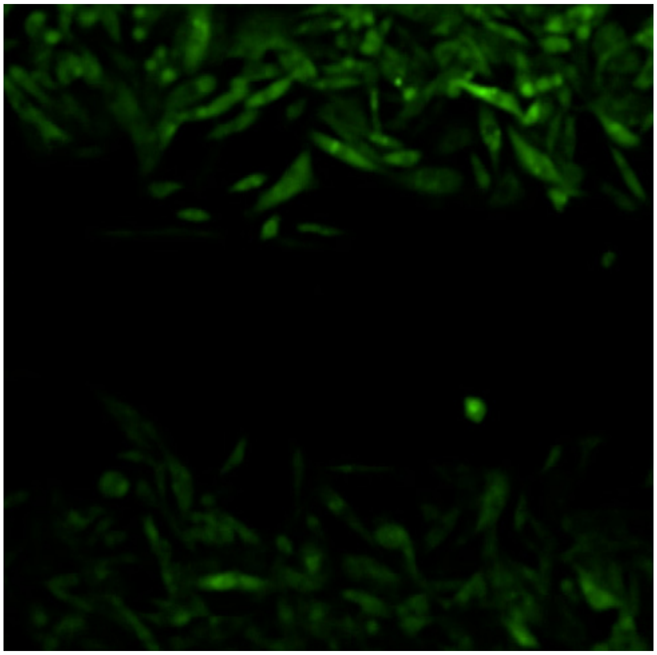

Supplementary Movie S1: The migration of Lenti-vector (MDA-MB-231) cells into the wound was monitored using a CellVoyager CV1000 confocal scanner system. The images were acquired every 0.5 hour for 28 hours.

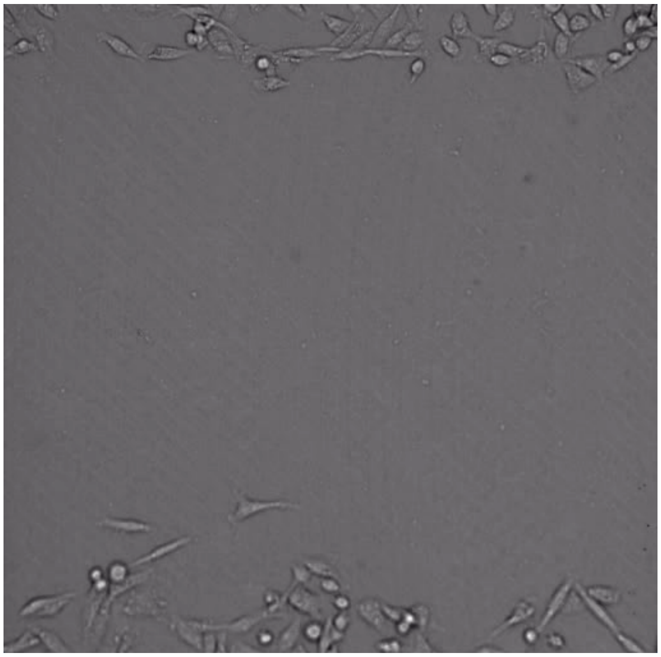

Supplementary Movie S2: The migration of Lenti-AGPAT9 (MDA-MB-231) cells into the wound was monitored using a CellVoyager CV1000 confocal scanner system. The images were acquired every 0.5 hour for 28 hours.

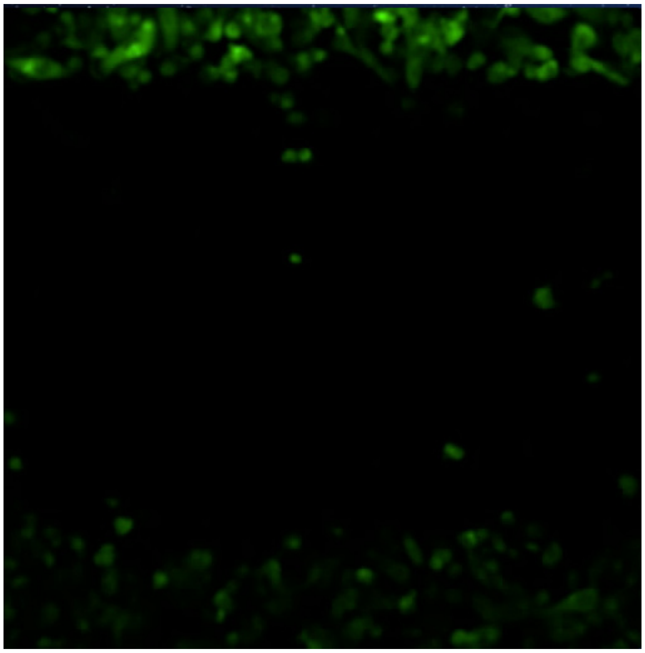

Supplementary Movie S3: The migration of Lenti-vector-GFP (MDA-MB-231) cells into the wound was monitored using a CellVoyager CV1000 confocal scanner system. The images were acquired every 0.5 hour for 35 hours.

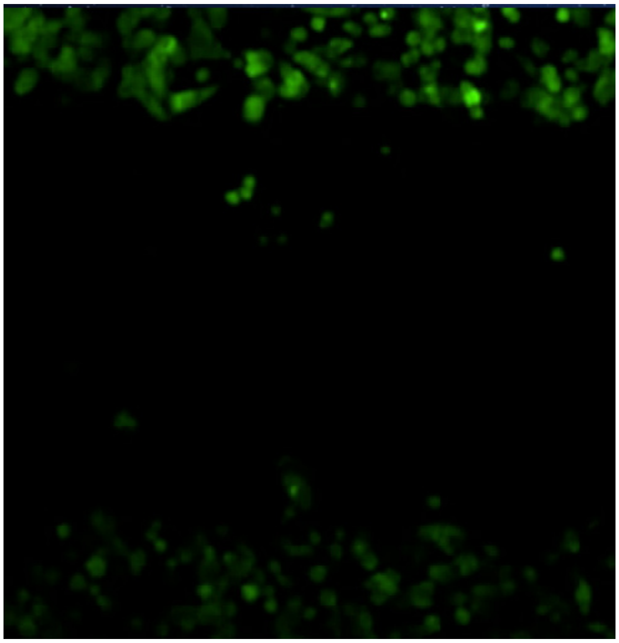

Supplementary Movie S4: The migration of Lenti-AGPAT9-GFP (MDA-MB-231) cells into the wound was monitored using a CellVoyager CV1000 confocal scanner system. The images were acquired every 0.5 hour for 35 hours.

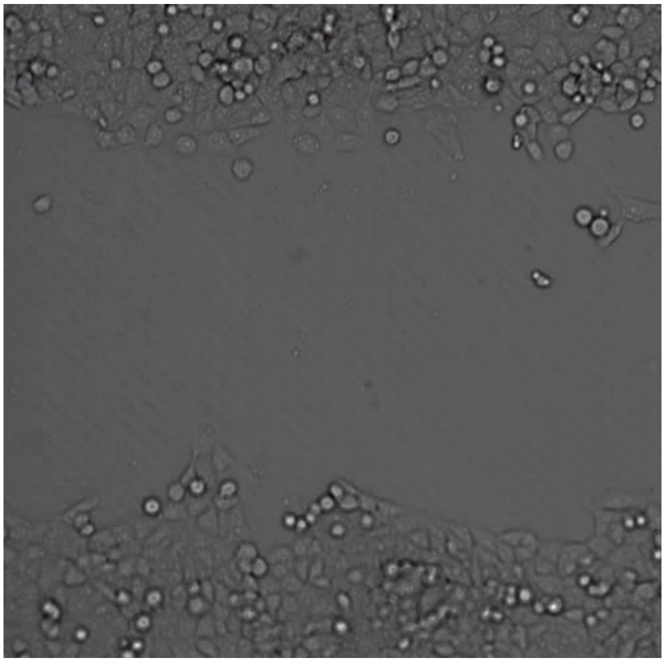

Supplementary Movie S5: The migration of Lenti-vector (MCF7/ADR) cells into the wound was monitored using a CellVoyager CV1000 confocal scanner system. The images were acquired every hour for 32 hours.

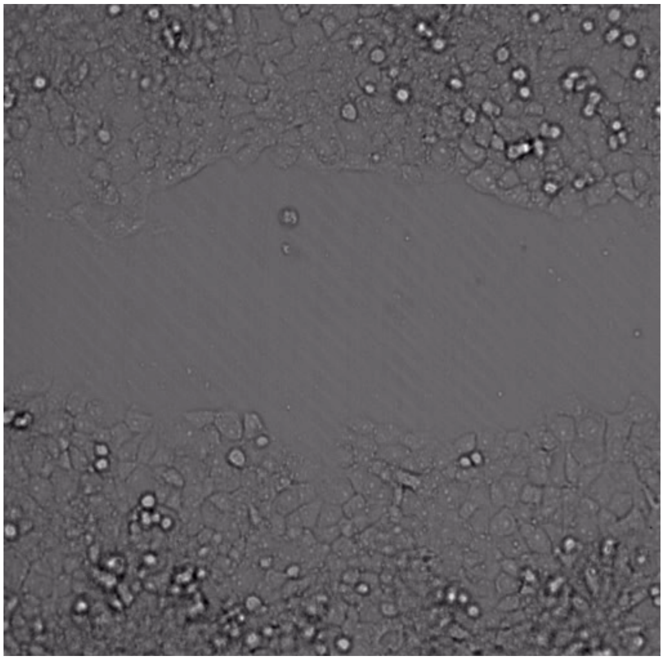

Supplementary Movie S6: The migration of Lenti-AGPAT9 (MCF7/ADR) cells into the wound was monitored using a CellVoyager CV1000 confocal scanner system. The images were acquired every hour for 32 hours.

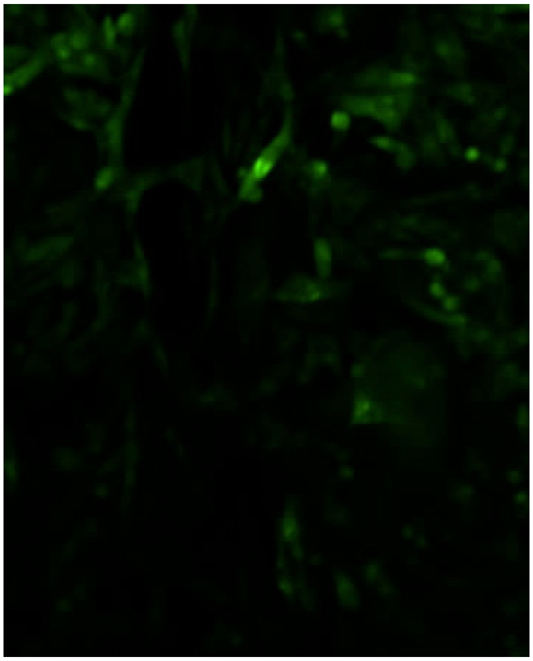

Supplementary Movie S7: The migration of Lenti-shRNA-vector (MDA-MB-231) cells into the wound was monitored using a CellVoyager CV1000 confocal scanner system. The images were acquired every 0.5 hour for 35 hours.

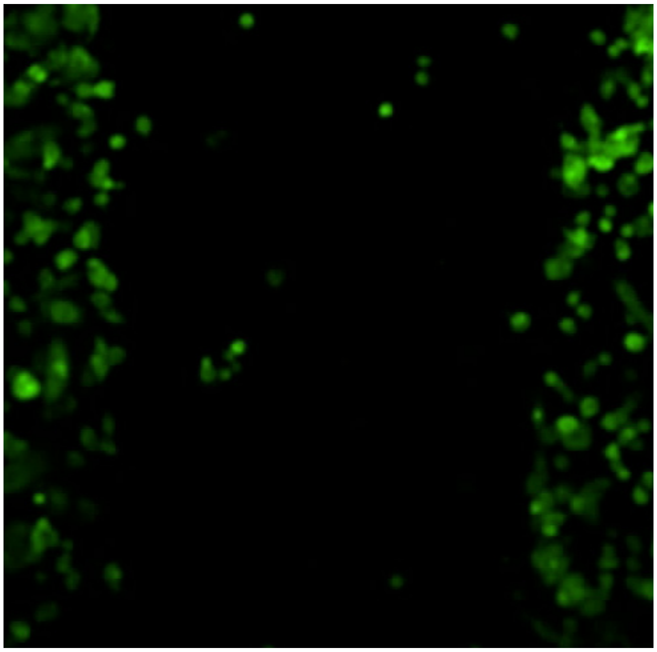

Supplementary Movie S8: The migration of Lenti-shRNA-ATP6V0C (MDA-MB-231) cells into the wound was monitored using a CellVoyager CV1000 confocal scanner system. The images were acquired every 0.5 hour for 35 hours.
